# Interactive Effect of Melatonin and UV-C on Phenylpropanoid Metabolite Production and Antioxidant Potential in Callus Cultures of Purple Basil (*Ocimum basilicum* L. var *purpurascens*)

**DOI:** 10.3390/molecules25051072

**Published:** 2020-02-27

**Authors:** Munazza Nazir, Muhammad Asad Ullah, Sadia Mumtaz, Aisha Siddiquah, Muzamil Shah, Samantha Drouet, Christophe Hano, Bilal Haider Abbasi

**Affiliations:** 1 Department of Biotechnology, Quaid-i-Azam University, Islamabad-45320, Pakistan; munazzabiotech2015@gmail.com (M.N.); asad_ullah8050@yahoo.com (M.A.U.); aisha_siddiquah@yahoo.com (A.S.); muzamilshah1989@gmail.com (M.S.); 2 Department of Botany, University of Azad Jammu &Kashmir, Muzaffarabad, Azad Kashmir 13230, Pakistan; 3 Department of Biotechnology, Women University of Azad Jammu &Kashmir Bagh, Azad Kashmir 12500, Pakistan; sadiamumtaz93@yahoo.com; 4 Laboratoire de Biologie des Ligneux et des Grandes Cultures (LBLGC), INRA USC1328, Université d’Orléans, 45067 Orléans CEDEX 2, France or or samantha.drouet@univ-orleans.fr (S.D.)

**Keywords:** elicitation, melatonin, phenylpropanoid metabolites, ultraviolet rays, antioxidant activities

## Abstract

The present study evaluated the interactive effect of melatonin and UV-C on phenylpropanoid metabolites profile and antioxidant potential of *Ocimum basilicum* L. Callus was treated with varying concentrations of melatonin and UV-C radiations for different time durations, either alone and/or in combination. Individual treatments of both UV-C and melatonin proved to be more effective than combine treatments. Results indicated that UV-C (10 min) exposure increased rosmarinic acid (134.5 mg/g dry weight (DW)), which was 2.3-fold greater than control. Chichoric acid (51.52 mg/g DW) and anthocyanin (cyanide 0.50 mg/g DW) were almost 4.1-fold, while peonidin was found 2.7-fold higher in UV-C (50 min) exposure. In the case of melatonin, 1.0 mg/L concentrations showed maximum rosmarinic acid (79.4 mg/g DW) accumulation; i.e., 1.4-fold more, as compared to the control. However, 2 mg/L melatonin accumulate chichoric acid (39.99 mg/g DW) and anthocyanin (cyanide: 0.45 mg/g DW and peonidin: 0.22 mg/g DW); i.e., 3.2, 3.7 and 2.0-fold increase, as compared to the control, respectively. On the other hand, melatonin-combined treatment (melatonin (Mel) (4 mg/L) + UV-C (20 min)) was proved to be effective in caffeic acid elicitation, which was 1.9-fold greater than the control. Furthermore, antioxidant potential was evaluated by both in vitro (DPPH, ABTS and FRAP assays) and *in cellulo* methods. Maximum in vitro antioxidant activity (DPPH: 90.6% and ABTS: 1909.5 µM) was observed for UV-C (50 min)-treated cultures. The highest in vitro antioxidant activity measured with the ABTS assay as compared to the FRAP assay, suggesting the main contribution of antioxidants from basil callus extracts acting through a hydrogen atom transfer (HAT) over an electron transfer (ET)-based mechanism. Cellular antioxidant assay was evaluated by production of ROS/RNS species using yeast cell cultures and further confirmed the protective action of the corresponding callus extracts against oxidative stress. Overall, both melatonin and UV-C are here proved to be effective elicitors since a positive correlation between the induced production of phenolic compounds, and *in cellulo* antioxidant action of basil callus extracts were observed.

## 1. Introduction

*Ocimum basilicum* L. var *purpurascens* (*Lamiaceae*) is native to tropical and subtropical zones, which has got importance due to its aromatic and ornamental attributes and is medicinally important due to the presence of volatile secondary metabolites like rosmarinic acid, flavonoids and anthocyanins [1]. Rosmarinic acid and anthocyanins are most significant caffeic acid esters which are abundantly synthesized in *Ocimum* spp., having various pharmacological and therapeutics properties [2,3,4,5,6]. These compounds are believed to have significant roles in stress-coping mechanisms and help plants to interact with their environment. The potential applications of these compounds as antioxidant molecules is of great important to humans [7,8]. The overexploitation and depletion of natural resources is the major concern of the world today. The recent developments in the genetics and biotechnology has stressed upon the use of in vitro cultures [9]. Major areas which have got a focus on plant tissue cultures are the breeding and genetics, model systems for plant genetics, physiology, biochemistry and pathology and, lastly, secondary metabolites production [10]. Fast proliferation of cell mass due to cell growth leads to high metabolic rates in cell cultures when compared to the intact differentiated plants. This is one of the most important advantages of cell cultures for secondary metabolites production [11]. Elicitation is among the most (cost)-effective strategy, which increases productivity of bioactive secondary metabolites [12]. Elicitors are extensively utilized for enhanced biosynthesis of plant metabolites in cell cultures by shortening the time of process for increased culture volume and high concentrations of products [13]. Biotic and abiotic are the two main classes of elicitors, based on their origin [14].

Melatonin (*N*-acetyl-5-methoxytryptamine) is an indole compound derived from serotonin [15,16]. The high range of melatonin’s biological applications has led it to be regarded as a multiregulatory molecule. These molecules act as biostimulators against stress conditions and regulators of plant growth and vegetative development, in flowering and fruit development and ripening [17,18,19]. Previous reports indicate that the exogenous application of melatonin increases antioxidants and phenolic compounds of wine and grape berries [20]. Likewise, it has also been suggested that melatonin at different concentrations increases the aromatic content and phenolic compounds accumulation in R. officinalis callus cultures [21]. Moreover, melatonin actively mitigate/counter the harmful effects of different abiotic stresses, such as UV radiations, drought, salinity, cold, heat and chemical toxicity [15]. Ultraviolet radiations act as an important abiotic component in tissue cultures that accelerates the biosynthesis of auxiliary metabolites [22]. The accumulation of UV-absorbing compounds, like phenolics, is the most prevalent defense mechanism and most frequent response by the plants [23]. Exposure of plants to the UV light stress enables their defense mechanism to stimulate and produce essential plant phytochemicals [24]. The antioxidant enzymatic biosynthesis which results due to the activation of plant defense mechanism leads to the amendment of cells to cope with the environment. These compounds’ productions act as a defensive barrier of plant cells from ROS (reactive oxygen species) produced to UV-induced stress responses [25,26]. Apart from the other UV types, UV-C radiation (range 190–280 nm) have verified to be more active in phenolics, flavonoids, glucosinolates and tocopherols biosynthesis [27].

Procedures of elicitation are sometimes combined to enhance efficacy of procedure. For instance, melatonin and different light regimes have synergistically improved the anti-inflammatory and antioxidant activities, as well as the silymarin content in callus cultures of *S. marianum* [28]. Ullah et al. verified the effect of UV-C radiation and melatonin on the synthesis of antioxidant and production antidiabetic metabolites in callus cultures of *Lepidium sativum* L. [29].

Therefore, the current study was designed to explore the effects of melatonin, UV-C and combined elicitations on the biosynthesis of phytochemicals in in vitro derived calli of purple basil and to evaluate their antioxidant potential. In future, elicitation with melatonin and UV-C would serve as a powerful technique to improve the rosmarinic acid, caffeic acid, chicoric acid and anthocyanin productions in cell cultures of purple basil.

## 2. Results and Discussion

### 2.1. Biomass Accumulation under Melatonin and UV-C Treatments

**Figure 1:**
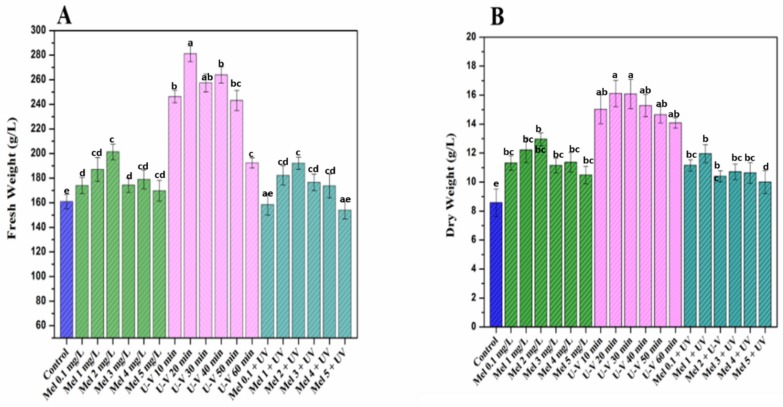
(**A**) Biomass (fresh weight) accumulation at optimized hormonal conditions under di*ff*erent treatments of UV-C, melatonin and melatonin + UV-C and (**B**) biomass (dry weight) accumulation at optimized hormonal conditions under different treatments of UV-C, melatonin and melatonin + UV-C. Values are means ± SE from triplicates. Columns with similar alphabets are not significantly different (*p* < 0.05) according to Tukey’s test.

The influence of melatonin and UV-C exposure time on growth of purple basil callus was assessed at various concentrations of elicitors for optimum growth and biomass production, as illustrated in Figure 1A, B. Results indicated an overall increase in biomass accumulation in purple basil under the influence of elicitor’s stress. However, highest accumulations of biomass (fresh weight (FW): 281.1 g/L and dry weight (DW): 16.4 g/L) was observed for UV-C (20 min) exposure, whereas UV-C (60 min) showed minimal response in callus growths, as evident from the least biomass yield (FW: 192.8 g/L and DW: 14.0 g/L). Significant decreases in biomass could be justified due to irreversible cell damage, triggered by longer duration of radiation exposure, resulting in cellular death [30,31]. However, in response to short-term exposure (20 min) to UV-C radiations, an increased yield of biomass was documented in *Vitis vinifera* cell cultures [32]. Similarly, Ullah et al. [29] reported that UV-C exposure for longer periods significantly reduced cell growth and biomass in callus cultures of *Lepedium satvium* L. This point was further elaborated and confirmed by different in vitro studies on medicinal plants in which the growth was stimulated on exposure to UV-C radiations [32,33]. Likewise, Anjum et al. [34] observed that there is an increase in biomass production when *Linum usitatissimum* L. cell cultures were subjected to UV-C (20 min) radiation treatment. Considerable results were achieved for the exogenous application of melatonin (Mel) at different concentrations. Maximum biomass (FW, 201.3 g/L and DW, 12.9 g/L) production was detected at 2 mg/L concentration of melatonin, while minimum (FW, 169.6 mg/L and DW, 10.4 g/L) was detected at 5 mg/L melatonin, indicating that low concentrations of Mel has positive effects on growth parameters of purple basil callus cultures. Higher melatonin concentrations inhibited biomass accumulation [35]. The higher concentration of melatonin might have led to ROS generation, leading to apoptosis of cells and, thus, inhibiting the cellular growth and proliferation [36]. The present findings coincide with the previously observed higher melatonin treatments’ inhibitory effects on biomass accumulation [29,36,37].

### 2.2. Influence UV-C radiations and Melatonin on Total Phenolic and Flavonoid Productions

**Figure 2:**
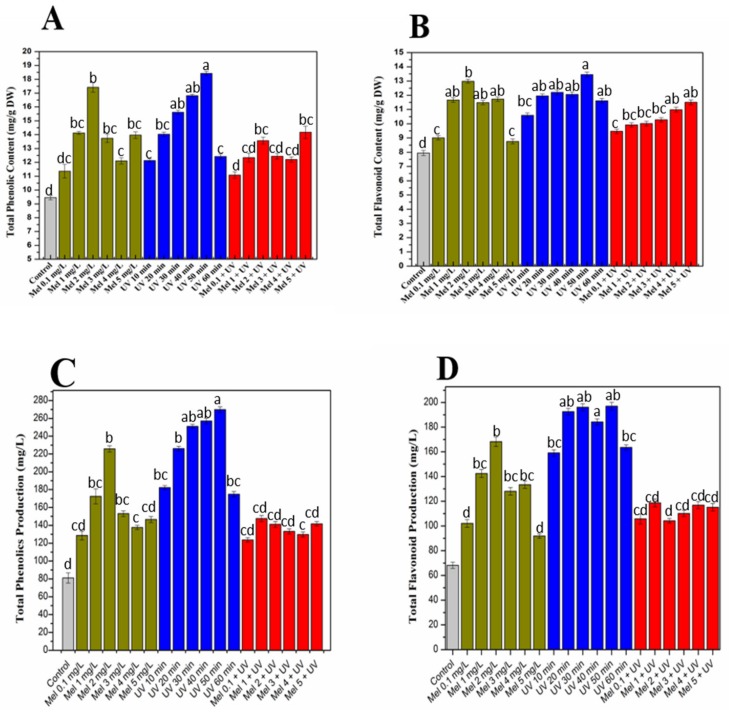
Total phenolic content (TPC), total flavonoid content (TFC) and their production at optimized hormonal conditions under different treatments of UV-C, melatonin and Mel + UV-C. (**A**) TPC (mg/g DW), (**B**) TFC (mg/g DW), (**C**) total phenolic production (TPP) (mg/L) and (**D**) total flavonoid production (TFP) (mg/L). Values are means ± SE from triplicates. Columns with similar alphabets are not significantly different (*p* < 0.05), according to Tukey’s test.

Elicitors have been seemed to increase the phenolic composition of purple basil callus cultures when elicited with melatonin, UV-C and Mel + UV-C (Figure 2A). Results indicated higher productions of flavonoid and phenolic in irradiated calli as compared to all other applied treatments and the control (Figure 2A–D). Maximum phenolic (total phenolic content (TPC): 18.4 mg/g DW and total phenolic production (TPP): 269.8 mg/L) and flavonoid (total flavonoid content (TFC): 13.4 mg/g DW and total flavonoid production (TFP): 196.9 mg/L) accumulation was detected in calli exposed to UV-C for 50 min, while the lowest values of TPC (12.1 mg/g DW), TPP (174.2 mg/L), TFC (10.5 mg/g DW) and TFP(159.1 mg/L) were found at 10-min exposure, compared to the rest of UV-C treatments. These results are in harmony with numerous reports in literature stating the potential role of UV-C radiation on different medicinal plants [26,27,29]. High phenolic production in response to UV-C exposure might be responsible for defensive mechanisms of purple basil, as it has been suggested that plants developed defensive mechanisms by synthesizing and storing UV-absorbing compounds to prevent damage by excessive UV radiations [38]. However, the mechanism of action is not clearly elucidated yet, but research has shown that UV stress is encountered in plants by modulating key enzymes productions, such as CHS (chalcone synthase), PAL (phenylalanine ammonia–lyase) and through the rapid accumulation of phenolic compounds [39,40,41]. Exogenous applications of melatonin affect plants’ physiological processes [42,43]. The effects of different melatonin concentrations on purple basil callus cultures were also studied for flavonoids and phenolics production. Melatonin (2 mg/L) enhanced total phenolic (TPC: 17.4 mg/g DW and TPP: 225.6 mg/L) and flavonoid (TFC: 12.9 mg/g DW and TFC: 168.1 mg/L) accumulation, while minimum TPC (11.3 mg/g DW and TPP: 128.6 mg/L), TFC (8.7 mg/g DW) and TFP (91.8 mg/L) were detected for 0.1 mg/L and 5 mg/L concentrations of melatonin, respectively. Our findings coincide with the study of Dawood and El-Awadi [44] stating the gradual increase in *Vicia faba* L. leaf tissues’ phenolic compounds by melatonin treatments. Comparable to current results, an increase in phenolic compounds in response to melatonin application in *Vigna radiata* L. was also observed [45]. Similarly, Ullah et al. [29] documented results in Lepidium sativum callus culture, which was also in harmony with present study findings. Based on biomass accumulation, UV-C (20 min) was applied with combinations of different concentrations of melatonin and further screened for phenolic and flavonoids accumulation. Maximum TPC (14.1 mg/g DW), TPP (143.7 mg/L), TFC (11.5 mg/g DW) and TFP (120.1 mg/L) were observed for Mel (5 mg/L) + UV-C (20 min). Minimum TPC (11.0 mg/g DW), TPP (123.6 mg/L), TFC (9.4 mg/g DW) and TFP (99.6 mg/L) were observed for Mel (0.1 mg/L) + UV (20 min). Current study results revealed that combined treatments of UV-C and melatonin enhanced phenolic and flavonoid profiles of purple basil callus cultures as compared to the control, but their alone treatments have more positive effects than a combination, suggesting that in basil cultures alone, treatments are more effective than a combination.

### 2.3. Quantification of Phenylpropanoid Metabolites Via HPLC

Elicitation strategies are used in in vitro production systems for increased production and accumulation of important metabolites [46]. Usually, chemical elicitation is attained by modulation through plant growth regulators, signaling mediators or progenitor moieties. Physical elicitation is caused by altering parameters like heavy metals, concentration, pressure, electric field, UV irradiation, pH and temperature [47]. There are many situations in which in vitro-derived cultures yield low secondary metabolites where enhanced production is required. This might be due to inhibitory effects of enzymes or because of the short stationary phase [48]. In the current study, phenylpropanoid metabolites quantification was done via HPLC. Rosmarinic acid (RA) is a major phenolic compound of purple basil. Elicitors applied individually and/or in combination significantly increased RA production in callus cultures compared to the control. Overall, UV-C elicited higher levels of RA as compared to rest of the treatments. Maximum accumulation of RA (134.5 mg/g) was observed against 10-min exposure to UV-C, while minimum production (110.8 mg/g) was observed for 50-min exposure. This increase might be due to enhanced phenylalanine ammonia lyase (PAL) and chalcone synthase (CHS) formations stimulated under UV exposure [49,50]. It has been observed that biosynthesis of phenylpropanoids is induced due to exposure with high light, temperature and UV irradiation [51]. Similar results were noted in terms of enhanced RA biosynthesis against exogenous applications of melatonin. Mel (1 mg/L) showed maximum accumulation (79.4 mg/g DW) of RA, whereas melatonin (4 mg/L) showed minimum accumulation of RA (65.9 mg/g DW) (Table 1). These results are in accordance with a study in which exogenous melatonin exposure produced variable physiological responses in *Lepidium sativum* [29] and *Silybum marianum* [28]. Duran et al. [52] concluded that melatonin application increased RA contents 5-fold more as compared to the control in sweet basil callus cultures. Combination of Mel (1 mg/L) + UV-C (20 min) showed 1.5-fold more RA accumulation than the control but lower when applied alone. Other medicinally important metabolites of purple basil, including chicoric acid and anthocyanins, were also enhanced by melatonin (2 mg/L) and UV-C (50 min) treatments. Stimulation of the synthesis of anthocyanins by UV radiation might be explained by the fact that they act as an absorbent in the UV region of the spectrum and, therefore, are capable of protecting plant cells from the harmful effects of UV [53]. Melatonin (2 mg/L) presented with enhanced accumulations of chicoric acid (39.9 mg/g DW) and anthocyanins (cyanidin 0.45 mg/g DW and peonidin 0.22 mg/g DW). Similarly, Sumaira et al. (2017) suggested the inducing role of melatonin in the biosynthesis of various phytochemicals [54]. Herein, similar observations were documented, which involve augmentation in the synthesis of phenolic compounds in calli exposed to melatonin [29,42,43,45]. A similar trend was observed for UV-C treatments. UV-C (50 min) showed maximum chicoric acid (51.52 mg/g DW) and anthocyanin (cyanidin 0.50 mg/g DW and peonidin 0.30 mg/g DW) accumulations, whereas UV-C (10-min) exposure downregulated their biosynthesis. Up-regulation of structural and regulatory genes involved during stress situations in anthocyanins biosynthesis could possibly be the reason behind its increased accumulation [55]. There are corresponding reports of anthocyanin formation in response to UV exposure in Arabidopsis [56], grape [57], carrot cells [58] and eggplant [59]. However, maximum caffeic acid elicitation was observed in response to the combination of Mel (4 mg/L) + UV-C (20 min). Exposure of plants to the UV light stress enables their defense mechanism to stimulate and produce essential plant phytochemicals [24]. However, it also induces generation of ROS, which rapidly reacts with different macromolecules, such as proteins and lipids, thus inducing mitochondrial membrane damage [60,61]. Melatonin actively mitigates/counters the harmful effects of UV radiations [15] by reducing electron leakage from mitochondria by stimulating the activities of the respiratory chain complexes [61,62], but in the current study, the effect is less when exposed to melatonin and UV-C together; this might be due to failure of melatonin concentrations to mitigate UV stress and their defense mechanism unable to produce other compounds. Applications of these elicitors could be an effective strategy for elicitation of certain secondary metabolites in purple basil callus cultures.

### 2.4. Antioxidant Activities of Purple Basil Callus Culture under UV-C light and Melatonin Treatments

**Figure 3:**
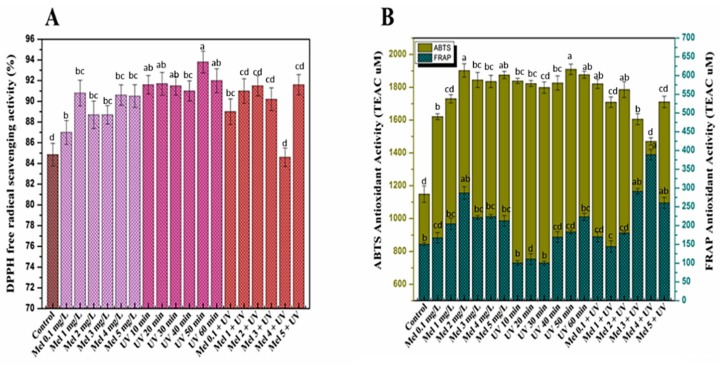
(**A**) % free radical scavenging and (**B**) antioxidant activities (ABTS and FRAP (TEAC µM)) in purple basil calli in response to different treatments of UV-C, melatonin and Mel + UV-C. Values are means ± SE from triplicates. Columns with similar alphabets are not significantly different (*p* < 0.05) according to Tukey’s test.

Plants responses to oxidative stress by causing sudden shifts in their metabolic pathways resulted in the reactive oxygen species formation which could damage plant cells, membrane lipid, protein and DNA. Plants also produce a variety of compounds, such as phenolic and flavonoids, that act as protecting mechanisms under oxidative stress [63]. Herein, purple basil callus cultures, subjected to different regimes of UV-C light exposure, melatonin and Mel + UV-C treatments, were subsequently tested for antioxidant activities by DPPH, ABTS and FRAP assays. DPPH assay assesses free radicals scavenging activity by reduction (DPPH to DPPH-H) through donating hydrogen atoms or electrons (Figure 3A). DPPH-based free radical scavenging activity is expressed in terms of percentage (%). Higher DPPH activities (90.6%, 93.8% and 91.6%) were noted by melatonin (1 mg/L), UV-C (50 min) and Mel (5 mg/L) + UV (20 min) treatments, respectively, compared to the control (84.8%). These findings are in accordance to Khan et al. [35], who reported high free radical scavenging activity in *Fagonia indica* when treated with melatonin (10 mg/L). Anjum et al. [34] concluded that the *Linum usitatissimum* cell cultures exhibited the highest DPPH activity on UV-C exposure for 10 min. FRAP (ferric-reducing antioxidant power) and ABTS (2,20-azino-bis-3-ethylbenzothiazoline-6-sulphonic acid) assays are generally classified as electron transfer (ET) and hydrogen atom transfer (HAT) reaction-based, respectively, though sometimes it is very difficult to distinguish between them [64]. The ability of an antioxidant in reduction of an oxidant probe can be measured by these assays [65]. ABTS and FRAP results were expressed in TEAC (Trolox C equivalent antioxidant capacity) and presented in Figure 3B. Melatonin (2 mg/L) showed the highest ABTS (1902.8 µM) and FRAP (286.8 µM) potential as compared to the rest of the melatonin treatments. Antioxidant enzymes activated by melatonin are the main reason behind its antioxidant activity [66]. This enzyme increases the efficiency of the mitochondrial electron transport chain and plays a crucial role in protecting plants from oxidative damage [67]. Similarly, UV-C (50 min) showed maximum ABTS (1909.5 µM) activity, whereas the highest FRAP (223.4 µM) activity was observed at UV-C (60 min). In combination, maximum ABTS (1820.5 µM) was observed at Mel (2 mg/L) + UV (20 min), and maximum FRAP (389.1 µM) was observed at Mel (4 mg/L) + UV (20 min) treatment. It is proposed that increased antioxidant and phenolics activities might be the protective response of purple basil callus to abiotic stress. The antioxidant behavior may be attributed to flavonoids such as anthocyanins, which also exhibit UV protective effects [68]. A study by Grzegorczyk et al. [69] confirmed compounds from plants tissues as a source of antioxidants such as carnosic acid, rosmarinic acid and carnosol. In short, elicitors exhibited good antioxidant activities when applied alone as compared to the combined treatments.

**Figure 4:**
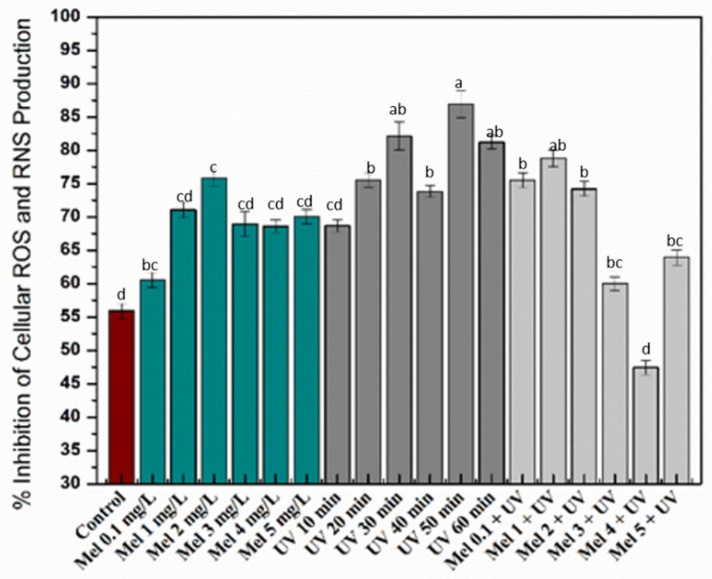
Percentage inhibition of cellular reactive oxygen/nitrogen species (ROS and RNS) production under different treatments of UV-C, melatonin and Mel + UV-C. Columns with similar alphabets are not significantly different (*p* < 0.05) according to Tukey’s test.

Oxidative stress is a state of disproportionation between reactive oxygen and/or nitrogen species (ROS/RNS, e.g., hydrogen peroxide, superoxide anion, peroxynitrite and hydroxyl radical) and antioxidant defense systems. The imbalance causes the oxidation of bio-macromolecules, such as DNA, enzymes, lipids and proteins. All this, if prolonged, results in the progression of various chronic degenerative conditions, including aging, coronary heart disease and cancer [64,70]. Here, cellular antioxidant assay confirmed the enhanced antioxidant capacity of the callus extracts resulting from the different treatments. Among the different treatments, melatonin (2 mg/L) and UV (50 min) presented the maximum cellular antioxidative protective effects on yeast cells (Figure 4). However, no synergism was observed when combining melatonin and UV treatment on the antioxidant activity of the resulting extracts.

**Figure 5:**
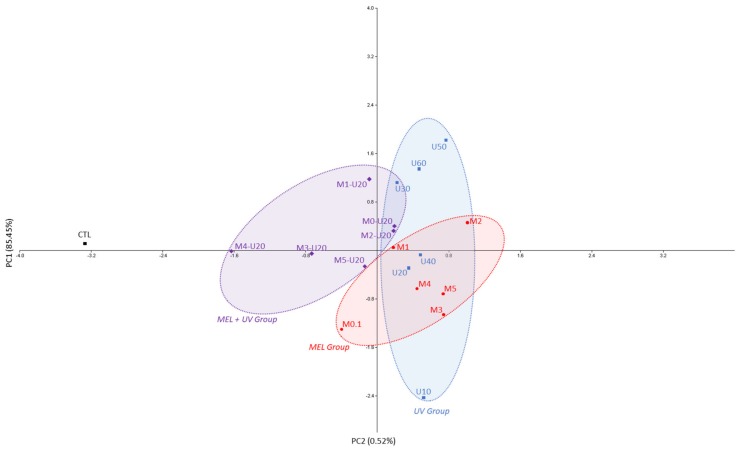
Principal component analysis (PCA) showing the impact of melatonin (MEL), UV-C (UV) and their combination on the phytochemical and antioxidant capacities of purple basil calli extracts. Variance of factor 1 (F1) = 85.45% and of factor 2 (F2) = 0.52%.

The phytochemical profiles and antioxidant capacities were subjected to principal component analysis. The resulting biplot representation accounts for 85.97% variance of factors 1 and 2 (F1 and F2) of the initial variability of the data (Figure 5). Loading scores of the first and second axes of the principal component analysis showing the impact of melatonin (MEL), UV-C (UV) and their combination on the phytochemical and antioxidant capacities of purple basil calli extracts are presented in Figures S1 and S2, respectively. Discrimination occurs mainly in the first dimension (PC1 axis explaining 85.45% of the variability) for the control versus treatment conditions resulting in a higher production of rosmarinic acid, chicoric acid, cyanidin and peonidin linked to higher DPPH, FRAP and cellular antioxidant capacities of the corresponding extracts. A clear discrimination between melatonin and UV-combined treatments was observed as compared to the single UV-C or melatonin treatments, thus evidencing an interactive action of these treatments on phytochemical production the and antioxidant capacity of purple basil calli extracts.

Calculation of Pearson’s correlation coefficient (PCC) between individual phytochemical profiles and different antioxidant assays evidenced strong significant correlation between rosmarinic acid, chicoric acid, cyanidin and peonidin, on the one hand, and DPPH, ABTS and cellular antioxidant assays on the other hand (Table 2). Note that caffeic acid was not correlated to rosmarinic acid and chicoric acid production, nor to antioxidant capacity of the extract. This could be related to the fact that caffeic acid is a direct precursor of both rosmarinic acid and chicoric acid, thus leading to a more complex relation. FRAP assay was not significantly or positively correlated to any of the phytochemicals here analyzed.

## 3. Materials and Methods

### 3.1. Seed Germination and Callogenesis

Leaf-derived explants were obtained from seedlings of in vitro cultured purple basil plantlets. Seeds were obtained from the NARC (National Agricultural Research Center, Islamabad, Pakistan). After external sterilization with distilled water (3 times), followed by 3 min ethanol (70%) and 1 min sodium hypochlorite treatment, seeds were placed in sterilized vials containing solid MS medium (Murashige and Skoog, 1962) supplemented with a carbon source (sucrose: 3%) and gelling agent (agar: 0.8%) at a pH of 5.6–5.7, prior to autoclave. For germination, all vials were kept in the growth chamber under a light cycle (16/8 h light/dark) and temperature (25 ± 2 °C). Callus cultures were established using leaf explants (0.5 cm^2^) from 28-days-old plantlets (in vitro-derived) cultured on MS media additionally supplied with NAA (2.5 mg/L), as optimized earlier by Nazir et al. [8]. Vials were shifted to the growth room for callogenesis. In order to get good-sized mass of callus for treatment by elicitors, callus was subcultured after every four weeks.

### 3.2. Elicitors Treatment on Callus Culture

#### 3.2.1. UV-C Treatment

UV-C radiation’s effect on growth of purple basil calli was evaluated by comparing with the control. UV-C lamp with 3 W/m^2^ radiation intensity (254 nm; Spectroline, model ZQJ-254, Hong Kong, China) was used as an ultraviolet light source. After inoculation on MS media, callus was radiated for different exposure time(s) with UV-C radiation from 15-cm distance. Calli were exposed to six (10, 20, 30, 40, 50 and 60 min) time durations of UV-C. Prior to exposure, the UV lamp was stabilized. In the growth room, the whole experiment was kept for four weeks. Calli without UV exposure was taken as the control. Harvesting of callus was carried out after four weeks to evaluate biomass and phytochemical accumulation.

#### 3.2.2. Melatonin Treatment

Melatonin effect on purple basil calli was evaluated by transferring fresh calli (1.0 g) from earlier subcultured callus on MS media having optimized hormonal dosage (NAA: 2.5 mg/L), additionally provided with diverse concentrations (0.1, 1.0, 2.0, 3.0, 4.0 and 5.0 mg/L) of melatonin. Melatonin-deprived media was taken as the control. For four weeks, the experiment was placed in a growth room. Calli were harvested after 28 days of inoculation for further analysis.

#### 3.2.3. Combined Melatonin + UV-C Treatment

Callus culture optimization results on changed UV-C exposure time showed optimum growth responses on UV-C (20 min) as compared to all other UV-C-applied treatments. To study combined effects of both elicitors, an optimized hormonal concentration (α-naphthalene acetic acid (NAA) 2.5 mg/L) calli (1.0 g) was used to inoculate on different melatonin concentrations (0.1, 1.0, 2.0, 3.0, 4.0 and 5.0 mg/L). Prior to inoculation, calli were exposed to UV-C (20 min) exposure at a fixed distance of 15 cm. For the control, callus without melatonin and UV-C treatment was used. Whole experiment was performed thrice and, after four weeks, harvested for further phytochemical analysis.

### 3.3. Preparation of Sample Extracts

Callus exposure to elicitor was harvested on Whatman filter paper after four weeks and left for one hour to remove extra water content. Fresh weight was determined for all treated callus cultures. Further, dry weight was measured after incubation for two days at 40 °C. Dry calli were grounded into a fine powder by using mortar and pistol. For extract preparations, protocol proposed by Nazir et al. [8] was followed.

### 3.4. Total Flavonoid and Phenolic Estimation

Revised procedure of Ul-Haq et al. [71] was followed to determine TFC. In short, mixture for the reaction contained callus extract (20 μL), potassium acetate (10 μL) and AlCl3 (10%), with 160 μL of dH_2_O. Absorption was noted at 415 nm by means of a microplate reader (Thermo Scientific Multiskan GO, Illkirch, France) after 30 min of incubation. TFC were then calculated using a standard quercetin (QE) curve and expressed as the equivalent of QE. Flavonoid production (TFP) was measured by multiplying TFC with respective dry weights:

Total flavonoid production (mg/L) = DW (g/L) × TFC (mg/g)(1)

Similarly, TPC were calculated by employing FC reagent through a revised method of Velioglu et al. [72]. Mixture contained 90 μL sodium carbonate and FC reagent (each), with 20 μL calli sample. Absorption was measured at 630 nm. For estimation of TPC, gallic acid was taken as standard calibration curve and expressed as equivalent of standard. TPP (total phenolic production) was determined as

Total phenolic production (mg/L) = DW (g/L) ×TPC (mg/g)(2)

### 3.5. Metabolic Quantification by HPLC Analysis

Phenylpropanoid metabolites quantification was done via HPLC supported by Hypersil PEP 300 C18 column (250 × 4.6 mm, 5 mm, Thermo Scientific, Illkirch, France), and the Alltech protection column (10 × 4.1 mm) was used for separation at 35 °C. The system of separation includes a Prostar 230 pump (Agilent Technology, Les Ulis, France), Metachem Degasit degasser (Agilent Technology, Les Ulis, France), Varian Prostar 410 autosampler (Agilent Technology, Les Ulis, France) and Varian Prostar 335 photodiode array detector (Agilent Technology, Les Ulis, France). It was attached with Galaxie software version 1.9.3.2 (Agilent Technology, Les Ulis, France). Compounds were detected at wavelengths of 320 nm (phenolic acids) and 520 nm (anthocyanins) by using the validated method described in the Nazir et al. [8] procedure. The quantification was performed based on the evaluation of respective retention times of reference standards of peonidin, rosmarinic acid, caffeic acid, cyanidin and chicoric acid obtained from Sigma-Aldrich (Sigma Aldrich, Saint Quentin Falavier, France). Every sample testing was done thrice, and results were expressed as mg/g DW of the sample.

### 3.6. Estimation of Antioxidant Potential

#### 3.6.1. DPPH Radical Scavenging Activity (%)

Free radical quenching activity DPPH (2,2-diphenyl-1-picrylhydrazyl) of the extracts was done by using the Abbasi et al. [73] protocol. Calli samples’ ability to scavenge free radicals was analyzed via mixing sample extracts of 20 µL with 180 µL of DPPH reagent and allowing it to incubate in the dark for 1 h. For the negative control, ascorbic acid final concentrations (05, 10, 20 and 40 µg/mL) and 180 µL of DPPH with 20 µL of DMSO were used. Solution absorbance was recorded at 517 nm with a microplate reader (Synergy II reader, BioTek Instruments, Colmar, France). DPPH activity was calculated using following formula:

% scavenging = 100 × (1 − AE/AD)(3)

Here, AE and AD denotes the absorbance with and without the sample addition at the 517 nm wavelength.

#### 3.6.2. Ferric-Reducing Antioxidant Power (FRAP) Assay

In order to evaluate the ferric-reducing antioxidant power, the Benzie and Strain [74] method with some modifications was used. In summary, FRAP solution (190 µL) (containing 2,4,6-Tri(2-pyridyl)-*s*-triazine (TPTZ, 10 mM); acetate buffer (300 mM) of pH 3.6 and 20-mM ferric chloride hexahydrate (FeCl_3_.6H_2_O); ratio 10:1:1 (*v*/*v*/*v*)) was added with samples (10 µL) and then kept at 25 °C for 15 min. Microplate reader (Synergy II reader, BioTek Instruments, Colmar, France) was used to measure absorbance at 630 nm. The entire assay was repeated three times. Antioxidant potential was expressed as the Trolox C equivalent antioxidant capacity (TEAC μM).

#### 3.6.3. Antioxidant ABTS Assay

ABTS (2,2-azinobis-3-ethylbenzthiazoline-6-sulphonic acid) assay was evaluated by the Tagliazucchi et al. [75] method with slight modifications. Briefly, the ABTS solution was prepared by mixing ABTS salt (7 mM) in equal proportion with potassium per sulphate (2.45 mM). This mixture was then incubated in the dark for 16 h. The solution absorbance was determined at 734 nm and adjusted to 0.7 before its use with the extracts. A mixture containing 10 µL of extract and 190 µL of the adjusted ABTS reagent solution was realized for each extract and incubated in the dark for 15 min at room temperature (25 °C) before reading the absorbance at 734 nm using a microplate reader (BioTek ELX800 Absorbance Microplate Reader, BioTek Instruments, Colmar, France). Each assay was realized in triplicate, and the antioxidant activity was expressed as TEAC.

#### 3.6.4. Cellular Antioxidant Assay

Nazir et al. [8] protocol, slightly modified, was followed for this assay. Reactive nitrogen and oxygen species production was detected using DHR-123 (dihydrorhodamine-123) fluorescent dye (Wolak et al., 2014) in cells through the oxidation of respective florescent products. Yeast cells’ overnight growth was observed with sample or DMSO (control cells) and then rinsed two times with PBS. The sediment was suspended in the presence of 0.4 µM DHR-123 with PBS and, for 10 min, incubated in the darkness at 30 °C. The signal was detected after rinsing with PBS by Bio-Rad Versa Floor fluorometer (λ_ex_ = 505 nm and λ_em_= 535 nm).

### 3.7. Statistical Analysis

Experimentation was done in a synchronized way, with three samples for each test and performed three times. Origin software (Windows v8.5) was used for statistical analysis and graphs. All values by using the Microsoft Excel program were expressed as means ± SD. Tukey’s multiple comparisons test was employed for calculating significant differences. One-way analysis of variance (ANOVA) with significant difference *p* < 0.05 was used to compare the means of different treatments. Principal component analysis and Pearson’s correlation coefficients were obtained using XL-STAT 2020 (Addinsoft, Paris, France).

## 4. Conclusions

In purple basil calli, metabolic variations were successfully induced and observed by exposure to stressful environmental conditions. The present study showed the promoting effects of UV-C, melatonin and melatonin + UV-C treatments for upgraded biosynthesis of phenylpropanoid metabolites in callus cultures of *Ocimum basilicum* L. var *purpurascens*. Overall, calli with applications of UV-C radiation (50 min) resulted in highest phenolic accumulation compared to melatonin (either alone or in combination with UV) and also showed remarkable in vitro-based antioxidant activities that might be due to caffeic acid and its derivatives, such as rosmarinic acid, chicoric acid and anthocyanins (cyanidin and peonidin). However, melatonin (2.0 mg/L)-treated cultures presented higher secondary metabolites production, and both in vitro and cellular antioxidant potential, than the rest of the melatonin treatments. Therefore, based on current findings, it is safe to say that use of elicitors such as UV-C and melatonin can adequately actuate phytochemicals in purple basil, which may be another alternative rather than hereditary modification.

## Figures and Tables

**Table 1:** Effects of melatonin, UV-C and Mel + UV-C on accumulation of phenylpropanoids in callus cultures of purple basil. Values are means of three independent replicates, and similar alphabets are not significantly different at *p* < 0.05.

Treatment	Conc.	Phenylpropanoid Compounds (mg/g DW)				
		Rosmarinic Acid	Chicoric Acid	Caffeic Acid	Cynadin	Peonidin
Control	NAA	56.30 ± 3.85 ^d,e^	12.33 ± 1.05 ^e^	0.28 ± 0.08 ^d^	0.12 ± 0.006 ^e^	0.11 ± 0.001 ^e^
Melatonin (mg/L)	0.1	62.27 ± 1.94 ^d,e^	32.63 ± 1.41 ^c^	0.52 ± 0.01 ^a^	0.36 ± 0.07 ^c^	0.18 ± 0.004 ^c^
	1	79.49 ± 2.52 ^c,d^	37.31 ± 1.89 ^bc^	0.47 ± 0.03 ^ab^	0.44 ± 0.04 ^ab^	0.19 ± 0.006 ^c^
	2	77.68 ± 3.04 ^c,d^	39.99 ± 0.55 ^b^	0.41 ± 0.06 ^bc^	0.45 ± 0.009 ^ab^	0.22 ± 0.002 ^b,c^
	3	74.10 ± 4.23 ^c,d^	36.98 ± 0.34 ^b,c^	0.46 ± 0.07 ^b^	0.41 ± 0.06 ^b^	0.20 ± 0.001 ^c^
	4	65.95 ± 3.14 ^d,e^	36.18 ± 1.14 ^b,c^	0.45 ± 0.04 ^b^	0.40 ± 0.02 ^b^	0.21 ± 0.003 ^c^
	5	78.25 ± 2.42 ^c,d^	37.75 ± 1.31 ^b,c^	0.44 ± 0.02 ^b^	0.42 ± 0.08 ^b^	0.21 ± 0.007 ^c^
UV-C (min)	10	134.01 ± 3.95 ^a^	37.01 ± 1.19 ^b,c^	0.44 ± 0.05 ^b^	0.42 ± 0.09 ^b^	0.21 ± 0.001 ^b,c^
	20	77.81 ± 2.77 ^c,d^	40.65 ± 0.88 ^b^	0.41 ± 0.03 ^b,c^	0.40 ± 0.01 ^b^	0.24 ± 0.009 ^b^
	30	82.32 ± 2.11 ^c,d^	43.34 ± 0.97 ^a,b^	0.36 ± 0.06 ^c^	0.49 ± 0.03 ^a^	0.25 ± 0.007 ^b^
	40	89.24 ± 4.62 ^c^	43.13 ± 1.05 ^a,b^	0.32 ± 0.02 ^c,d^	0.50 ± 0.06 ^a^	0.25 ± 0.003 ^b^
	50	110.8 ± 6.39 ^b^	51.52 ± 2.57 ^a^	0.29 ± 0.08 ^c,d^	0.50 ± 0.02 ^a^	0.30 ± 0.005 ^a^
	60	97.61 ± 1.48 ^b,c^	39.77 ± 0.78 ^b^	0.041 ± 0.01 ^b,c^	0.45 ± 0.08 ^ab^	0.23 ± 0.002 ^b,c^
Melatonin + UV-C	0.1 + 20	74.94 ± 2.35 ^c,d^	41.40 ± 0.72 ^b^	0.42 ± 0.01 ^b,c^	0.46 ± 0.04 ^a,b^	0.24 ± 0.005 ^b^
	1 + 20	86.26 ± 1.43 ^b,c^	42.96 ± 0.79 ^b^	0.40 ± 0.05 ^b,c^	0.43 ± 0.01 ^a,b^	0.23 ± 0.002 ^b,c^
	2 + 20	75.65 ± 2.55 ^c,d^	39.99 ± 1.58 ^b^	0.41 ± 0.08 ^b,c^	0.44 ± 0.03 ^a,b^	0.24 ± 0.008 ^b^
	3 + 20	53.93 ± 3.28 ^d,e^	30.88 ± 1.07 ^c^	0.50 ± 0.01 ^a,b^	0.36 ± 0.05 ^c^	0.18 ± 0.004 ^c^
	4 + 20	49.56 ± 2.82 ^e^	23.67 ± 0.98 ^d^	0.54 ± 0.07 ^a^	0.28 ± 0.04 ^d^	0.14 ± 0.006 ^e^
	5 + 20	68.49 ± 6.03 ^d^	33.76 ± 1.43 ^bc^	0.47 ± 0.09 ^a,b^	0.38 ± 0.07 ^c^	0.19 ± 0.005 ^c^

**Table 2:** Correlation analysis using Pearson’s correlation coefficient (PCC).

Variables	Ros-A	Chi-A	Caff-A	Cyan	Peon	DPPH	ABTS	FRAP	cRO/NS
Ros-A									
Chi-A	0.613 **								
Caff-A	−0.412 ^ns^	−0.203 ^ns^							
Cyan	0.575 **	0.959 ***	−0.152 ^ns^						
Peon	0.622 **	0.965 ***	−0.331 ^ns^	0.895 ***					
DPPH	0.669 **	0.792 ***	−0.337 ^ns^	0.760 ***	0.792 ***				
ABTS	0.546 *	0.873 ***	−0.047 ^ns^	0.908 ***	0.780 ***	0.686 **			
FRAP	−0.546 *	−0.352 ^ns^	0.302 ^ns^	−0.253 ^ns^	−0.437 ^ns^	−0.425 ^ns^	−0.130 ^ns^		
cRO/NS	0.626 **	0.870 ***	−0.545 *	0.799 ***	0.896 ***	0.767 ***	0.681 **	−0.554 **	

Ros-A: rosmarinic acid, Chi-A: chicoric acid, Caff-A: caffeic acid, Cyan: cyanidin, Peon: peonidin, DPPH: free radical scavenging activity determined by DPPH assay, ABTS: ABTS antioxidant assay, FRAP: FRAP antioxidant assay and cRO/NS: cellular antioxidant assay (inhibition of ROS and RNS production in yeast cells). Significance level: * *p* < 0.05, ** *p* < 0.01, *** *p* < 0.001 and ns: not significant.

## Supplementary Materials

The following are available online: Figure S1: Loading scores of the first axis of the principal component analysis showing the impact of melatonin (MEL), UV-C (UV) and their combination on the phytochemical and antioxidant capacities of purple basil calli extracts. Figure S2: Loading scores of the second axis of the principal component analysis showing the impact of melatonin (MEL), UV-C (UV) and their combination on the phytochemical and antioxidant capacities of purple basil calli extracts.

## Abbreviations

ABTS	2,2-Azinobis 3-ethylbenzthiazoline-6-sulphonic acid
CA	Caffeic acid
CHS	Chalcone synthase
DHR-123	Dihydrorhodamine-123
DMSO	Dimethyl sulfoxide
DPPH	2,2-Diphenyl-1-picrylhydrazyl
DW	Dry weight
ET	Electron transfer
FRAP	Ferric-reducing antioxidant power
FW	Fresh weight
HAT	Hydrogen atom transfer
HPLC	High-performance liquid chromatography
Mel	Melatonin
MS	Murashige and Skoog
NAA	α-naphthalene acetic acid
PAL	Phenylalanine ammonia-lyase
RA	Rosmarinic acid
ROS	Reactive oxygen species
RNS	Reactive nitrogen species
TEAC	Trolox C equivalent antioxidant capacity
TFC:	Total flavonoid content
TFP	Total flavonoid production
TPP	Total phenolic production
TPC	Total phenolic content
UV-C	Ultraviolet radiation C
