# Supplementary material for: Interactive Effect of Melatonin and UV-C on Phenylpropanoid Metabolite Production and Antioxidant Potential in Callus Cultures of Purple Basil (Ocimum basilicum L. var purpurascens)
Source: Molecules. 2020 Feb 27;25(5):1072. doi: 10.3390/molecules25051072 (PMC7179200; doi:10.3390/molecules25051072)
Supplement: Supplementary file 1 [file molecules-25-01072-s001.pdf]

Supplementary Data;

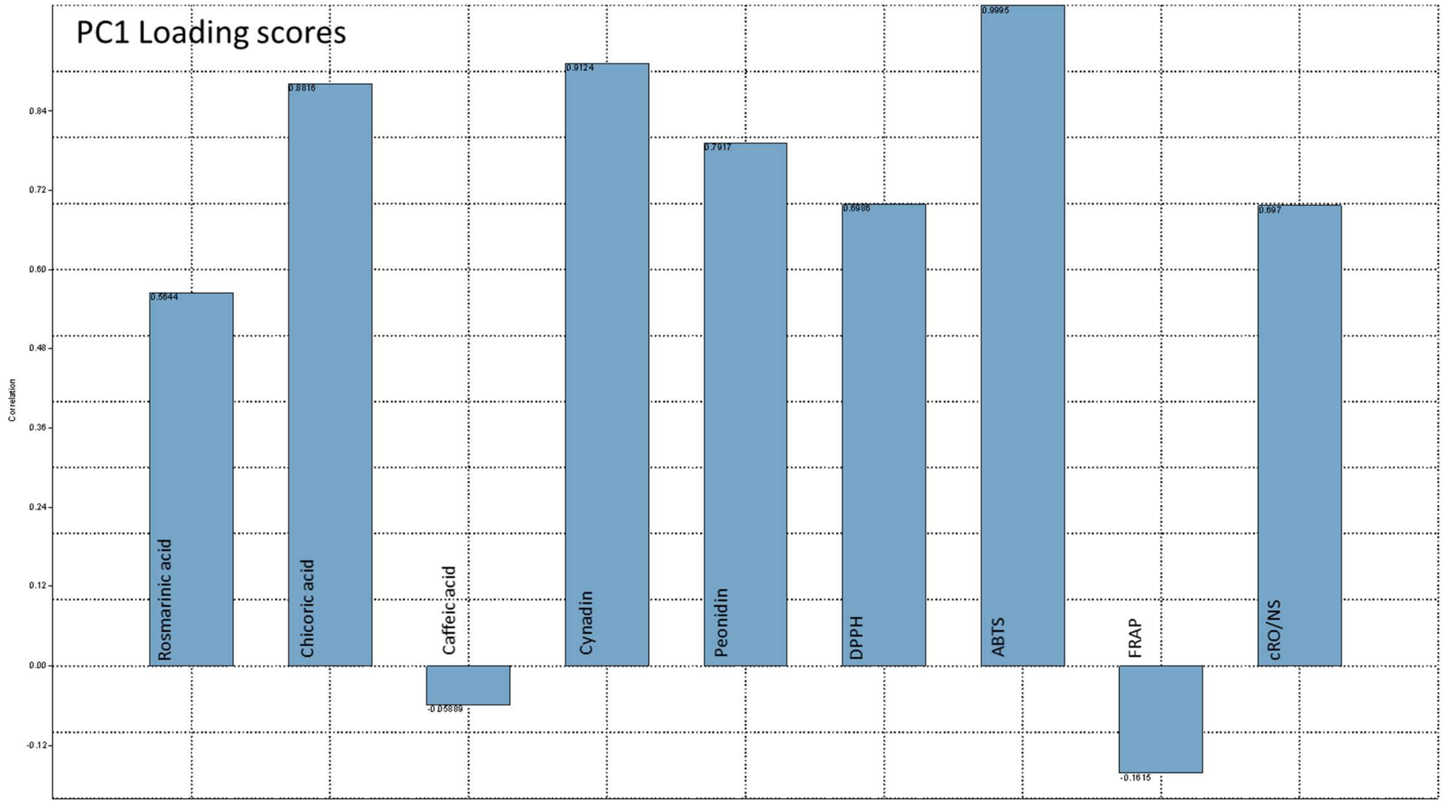

S1: Loading scores of the first axis of the principal component analysis showing the impact of melatonin (MEL), UV-C (UV) and their combination on the phytochemical and antioxidant capacities of purple basil calli extracts

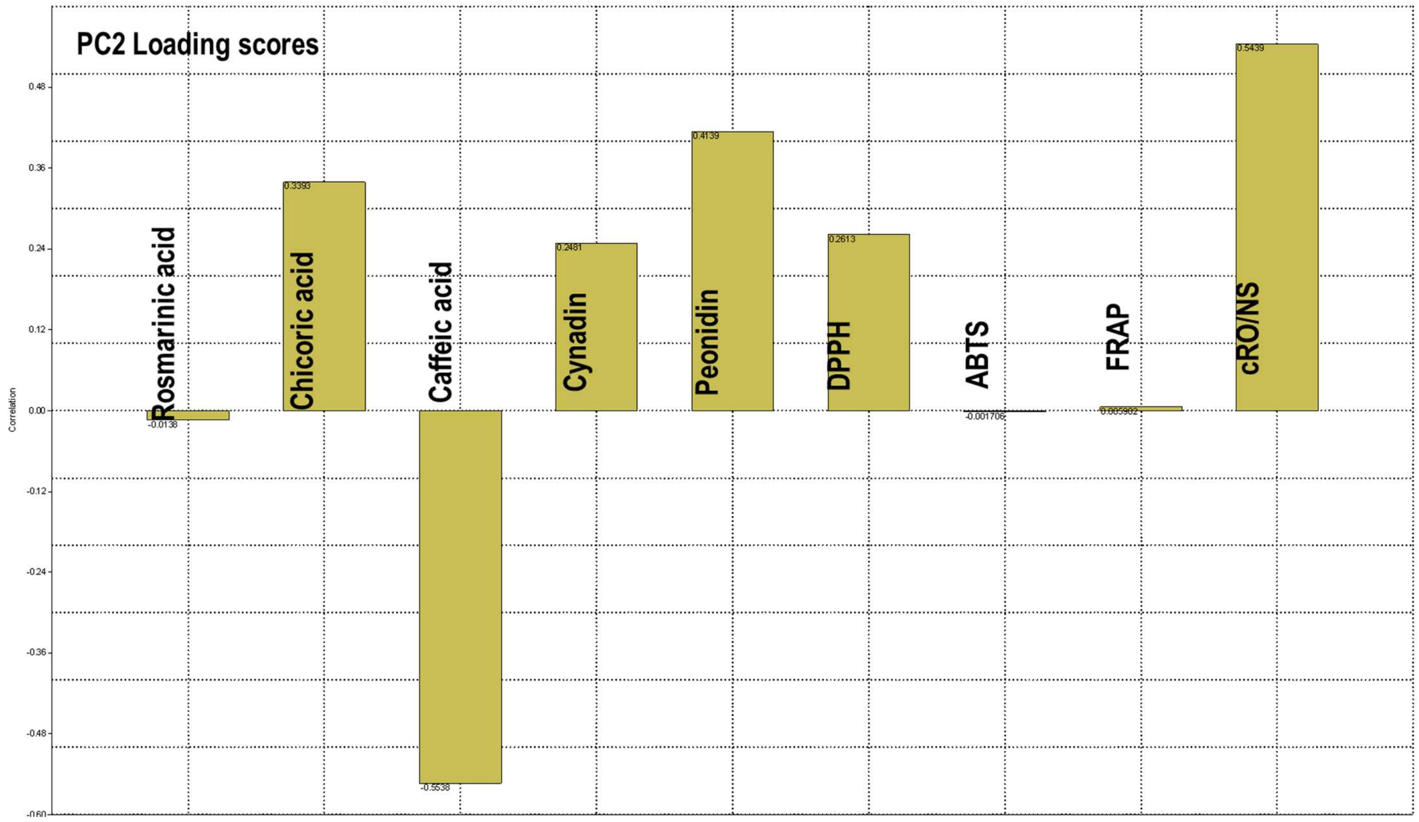

S2: Loading scores of the second axis of the principal component analysis showing the impact of melatonin (MEL), UV-C (UV) and their combination on the phytochemical and antioxidant capacities of purple basil calli extracts.
